# Thickness and Volume of Epicardial Adipose Tissue in Relation to Stiffness and Elasticity of Aorta Assessed by Computed Tomography Angiography

**DOI:** 10.3390/biomedicines11061617

**Published:** 2023-06-01

**Authors:** Paweł Gać, Wojciech Hajdusianek, Aleksandra Żórawik, Piotr Macek, Małgorzata Poręba, Rafał Poręba

**Affiliations:** 1 Department of Population Health, Division of Environmental Health and Occupational Medicine, Wroclaw Medical University, Mikulicza-Radeckiego 7, PL 50-368 Wroclaw, Poland; 2 Centre of Diagnostic Imaging, 4th Military Hospital, Weigla 5, PL 50-981 Wroclaw, Poland; 3 Department of Internal Medicine, Occupational Diseases, Hypertension and Clinical Oncology, Wroclaw Medical University, Borowska 213, PL 50-556 Wroclaw, Poland; 4 Department of Paralympic Sports, Wroclaw University of Health and Sport Sciences, Witelona 25a, PL 51-617 Wroclaw, Poland

**Keywords:** aortic distensibility, aortic stiffness index, aortic strain, coronary computed tomography angiography, epicardial adipose tissue

## Abstract

Purpose. The aim of the study was to assess the importance of the measurements of thickness and volume of epicardial adipose tissue (EAT) in coronary computed tomography angiography (CCTA) as a predictive factor of increased stiffness and impaired elasticity of aorta. Methods and materials. The study involved a group of 97 patients (63.48 ± 8.50 years). In accordance with the medians of epicardial adipose tissue (EAT) parameters, aortic elasticity and stiffness parameters, patients were divided into subgroups: EAT thickness median 9.40 mm, EAT volume median 61.95 mL, EAT thickness index 5.08 mm/m^2^ and EAT volume index 34.33 mL/m^2^. Results. The mean coronary artery calcium score was 162.24 (±317.69). The mean aortic stiffness index was 4.18 (±0.81). The assessed mean aortic elasticity parameters were 3.29% (±2.37) and 0.12 cm^2^/dyn (±0.09) for strain and distensibility, respectively. A positive linear correlation was observed between EAT parameters and aortic stiffness (0.21), volume (0.51), thickness index (0.24), volume index (0.55) and, for aorta elasticity, a negative linear correlation between the following EAT parameters was observed: thickness (−0.32 and −0.30), volume (−0.49 and −0.48), thickness index (−0.34 and −0.31), volume index (−0.51 and −0.49) and aortic elasticity parameters (aorta strain and aorta distensibility, respectively). Conclusion. The study showed that CCTA illustrates a relationship between the parameters of EAT and an increased stiffness of the aorta, while the most predictive factor of stiffness was the volume index.

## 1. Introduction

Coronary computed tomography angiography (CCTA), according to the European Society of Cardiology guidelines, can by used as a non-invasive way to evaluate lumen and the walls of coronary arteries by means of intravenous contrast agents. It provides high accuracy in the detection of coronary stenose; since not every visualized stenosis is functionally significant (and not always cause ischemia), further testing is usually conducted after stenosis detection in CCTA. The CCTA examination provides valuable prognostic information. The use of CCTA, in addition to the standard care of patients with stable chest pain, resulted in a lower rate of death from coronary artery disease (CAD). During the diagnosis of CAD, a CCTA is preferred in patients presenting a lower likelihood of CAD and not previously diagnosed with CAD. CCTA can find subclinical atherosclerosis in coronary arteries, and it can be used to identify those patients who are at risk [1,2,3,4,5].

CCTA can be used as a valuable tool to assess coronary arteries as described above. However, during the examination, it is possible to obtain interesting results that can indicate other diseases or cardiovascular risk factors. CCTA can be used to evaluate ablation strategies for patients with atrial fibrillation (AF) recurrences, despite pulmonary vain isolation. The measuring, in CCTA, of the left atrial appendage volume, which was considered as a potential source of AF triggers, was corelated to the left atrium volume and was a predictor of recurrence after cryoballoon ablation [6]. CCTA may be of assistance in the detection of early cardiac allograft vasculopathy in heart-transplanted patients by analysis of the coronary wall volume–length ratio, the wall burden and the proportion of fibrotic tissue. Those three parameters may aid in the early cardiac allograft vasculopathy that was not detected in invasive coronary angiography [7]. CCTA may be a useful technology during the diagnosis of a patient with spontaneous coronary artery dissection. In one study, features such as abrupt luminal stenosis, intramural hematoma, tapered luminal stenosis, and dissection were classified in CCTA [8]. CCTA can be used to measure aortic valve calcification (AVC), which is associated with cardiovascular events. Usually, the quantification of AVC is performed with the Agatston method with non-contrast enhanced ECG-gated computed tomography images. In patients that require transcatheter aortic valve implantation (TAVI), CCTA is routinely performed and the assessment of AVC and conducting of a pre-TAVI CCTA would require a two separate images; therefore, quantifying AVC during CCTA would enable to obtain all the information during one examination and reduce patient radiation exposition [9]. CCTA can be an important way to evaluate congenital non-coronary left heart abnormalities [10]. For example, during CCTA, various valve variants can be detected and their morphology evaluated which helps in the planning of appropriate treatment [11].

Epicardial adipose tissue (EAT) is a structure of increasing interest. Its role varies from the protection of nearby myocardium through its brown-fat thermogenic function to injuring through the paracrine secretion of cytokines. The amount of EAT is a modifiable risk factor of cardiovascular diseases. Cross-sectional studies correlated EAT expansion with the development of CAD. EAT is also thought to play a role in pathology of AF [12,13]. The amount of EAT is higher in obese patients whose diet contains higher amounts of fat. Disproportionate EAT may have lipotoxic effects due to the fatty infiltration of myocardium, because cardiomyocyte fat storage capacity is limited [14]. EAT, in the vasocrine mechanism, may release cytokines to the vasa vasorum of the coronary arterial wall, in which they will interact with the small vessels’ endothelium [15]. Due to its importance, some studies developed methods to automate EAT segmentation and quantification from CCTA scans with the aid of a neural network, to avoid time-consuming manual assessment and inter-investigator variability [16,17]. It was also suggested that EAT is associated with dyslipidemia [18]. EAT pro-inflammatory activity was reduced in intensively training athletes, which may be associated with a decreased risk of CVD events in this population [19]. EAT has also been associated with the development of atrial myopathy contributing to AF, ventricular hypertrophy and impaired relaxation and filling, which contribute to heart failure with preserved ejection fraction. Furthermore, EAT has also been related to hypertension and metabolic syndrome [20]. In patients with heart failure with preserved ejection fraction, EAT accumulation was associated with a worse hemodynamic profile [21]. EAT biologic activity was associated with coronary plaque vulnerability [22]. EAT was associated with the presence of non-calcified and mixed coronary plaques in groups of patients with coronary artery stenosis [23]. The relationship between the pro-inflammatory activity of EAT and the severity of coronary artery calcification may be a pathophysiological link explaining the increased cardiovascular mortality during COVID-19 [24]. Higher EAT was correlated with slowed conduction in cardiomyocytes. An increased amount of EAT increases atrial conduction heterogeneity and potentially contributes to AF [25]. EAT may be an independent risk factor for AF [26].

Different aortic stiffness and elasticity measurements methods, with a brief description of their importance, were described in our previous article [27] and the most relevant findings in the context of this study are as follows. Aortic stiffness was found to be a predictor of CAD [28], increased epicardial adipose tissue [29] and increased systolic blood pressure [30]. Reduced ascending aortic elasticity was found in the prehypertension group [31]. Aortic stiffness assessment was found to be a method to predict subclinical atherosclerosis [32].

The aim of the study was to determine the importance of the measurements of thickness and volume of epicardial adipose tissue in coronary computed tomography angiography as a predictive factor of increased stiffness and impaired elasticity of aorta.

## 2. Material and Methods

### 2.1. Calculation of Group Size, and Inclusion and Exclusion Criteria of the Study

The current study was performed as part of the project entitled: “Cardiovascular health and risk markers assessed by diagnostic imaging in patients with hypertension and obstructive sleep apnea”. Group size was determined using a sample size calculator. The selection conditions were as follows: population size—2.8 million, fraction size—0.5, maximum error—10%, confidence level—95%. The required minimum size of the study group was 97. Inclusion criteria were age ≥ 18, indication to coronary CT angiography and willingness to participate in the project. Based on the inclusion criteria, 106 patients were qualified for the study. The exclusion criteria were insufficient quality of the coronary CT angiography (2), previously diagnosed ischemic heart disease (4), previous stroke (2), type 2 diabetes (3), chronic kidney disease (1) and hypothyroidism (1). A total of 9 patients were disqualified from the study based on the presence of one or more exclusion criteria.

### 2.2. Study Group Description

There were 97 participants in the study: 48 (49.5%) men and 49 (50.5%) women. The mean age of participant was 63.48 (±8.50). The mean height and mean body mass were 1.67 m (±0.07 m) and 74.16 kg (±11.03 kg), respectively, therefore mean body mass index was 25.97 kg/m^2^ (±3.81 kg/m^2^). The calculated mean body surface area was 1.82 m^2^ (±0.15 m^2^). Based on anthropometric measurements, the participants were divided into ones with normal weight and ones with overweight or obesity. The number of participants presenting normal weight was 46 (47.4%), and with overweight or obesity it was 51 (52.6%). Participants presented a mean systolic blood pressure 143.20 mmHg (±7.78 mmHg) and mean diastolic blood pressure 88.40 mmHg (±5.66 mmHg). Patients included in the study presented various indications of coronary artery computed tomography (CCTA); among them, 68 (70.1%) of patients had a suspicion of chronic coronary artery diseases, 38 (39.2%) of patients presented a chest pain, 37 (39.1%) had a low-intermediate CAD risk, 26 (26.8%) were at risk of numerous factors, 13 (13.4%) of patients had an inconclusive exercise test and a further 8 (8.2%) of participants had a non-diagnostic exercise test. Two participants (2.1%) presented regional wall motion abnormalities of the left ventricle and one (1%) had a sudden cardiac death history in the family. The detailed summary is presented in Table 1.

### 2.3. Criteria for Separating the Subgroups—Medians of Studied Variables of Epicardial Fat Thickness and Aortic Stiffness and Elasticity

During the study, participants were divided into subgroups based on the medians of epicardial adipose tissue parameters, aortic elasticity and stiffness parameters. The median of aortic stiffness index was 4.09 and medians of parameters used to assess elasticity were 2.67% for aortic strain and 0.10 cm for aortic distensibility. The division of subgroups based on EAT was based on EAT thickness median 9.40 mm, EAT volume median 61.95 mL, EAT thickness index 5.08 mm/m^2^ and EAT volume index 34.33 mL/m^2^.

### 2.4. Methodology

Epicardial adipose tissue (EAT) was measured using the syngo.CT postprocessing application (Siemens Healthineers, Erlangen, Germany). Epicardial adipose tissue thickness (EAT thickness) was measured based on multiplanar reconstruction (MPR) in the short axis of the left ventricle, at the level of the mid-ventricular layer, halfway along the right ventricular free wall. The volume of epicardial adipose tissue (EAT volume) was measured in the native phase using a dedicated research prototype, i.e., Cardiac Risk Assessment (Siemens Healthineers, Erlangen, Germany), in accordance with the manufacturer’s instructions. Measurements were made semi-automatically with manual correction of EAT contours which were previously generated automatically by this tool. In addition, indexed values for thickness and volume of EAT (EAT thickness index and EAT volume index) were calculated by dividing the measured values by body surface area (BSA). Thanks to the use of a dedicated Cardiac Risk Assessment prototype (Siemens Healthineers, Erlangen, Germany), in the case of good quality CCTA images and an efficient diagnostic station, obtaining all EAT parameters included in the study took only a few minutes.

The aortic diameter (Ao diameter) was measured in the MPR reconstruction in a cross-section that was perpendicular to the long axis of the vessel and approximately 3.0 cm above the aortic valve ring. Measurements were made considering the phase of the heart cycle monitored by electrocardiography. Aortic systolic diameter was measured during full aortic valve opening, and aortic diastolic diameter was measured at the top of the R-wave of the recorded ECG. Aortic stiffness was assessed based on aortic stiffness index calculated on basis of the following mathematical formula (Equation (1)):

(1) $$Ao\;stiffness\;index=\ln\left(\frac{\frac{systolic\;blood\;pressure}{diastolic\;blood\;pressure}}{\frac{Ao\;systolic\;diameter-Ao\;diastolic\;diameter}{Ao\;diastolic\;diameter}}\right)$$

Aortic elasticity was assessed based on aortic strain and aortic distensibility in which (Equations (2) and (3)):

(2) $$Ao\;strain=\left(\frac{Ao\;systolic\;diameter-Ao\;diastolic\;diameter}{Ao\;diastolic\;diameter}\right)*100$$

(3) $$Ao\;distensibility=\frac{2x\;Ao\;strain}{systolic\;blood\;pressure-diastolic\;blood\;pressure}$$

### 2.5. Statistical Analysis

Statistical analysis was performed with the application “Dell Statistica” (Dell Inc., Round Rock, TX, USA). Quantitative variables were presented as arithmetic means ± standard deviations. The distribution of variables was determined by the Shapiro–Wilk test. For normally distributed quantitative variables, the *t*-test was used to test the hypotheses. For quantitative variables with no normal distribution, the Mann–Whitney U test was used to test the hypotheses. Qualitative variables were presented as numbers and percentages. To establish the relationship between the studied variables, a correlation analysis was performed. Moreover, evaluations of the test accuracy were performed based on the ROC (receiver operating characteristic) curve analysis. The results at the level of *p* < 0.05 were considered statistically significant.

## 3. Results

### 3.1. Coronary Computed Tomography Angiography Results and Measured Parameters of EAT and Aorta

Table 2 presents the results of the coronary computed tomography angiography parameters in the study group. The mean coronary artery calcium score among participants was 162.24 (±317.69). Among the participants subjected to CCTA, 31 (31.9%) presented as practically non-existent, 23 (23.7%) as minimal, 15 (15.5%) as mild, 16 (16.5%) as moderate and 12 (12.4%) as being at high coronary artery disease risk. Similarly, based on the coronary artery disease reporting and data system (CAD-RADS) in which 0 represents absence of CAD and five total occlusions of coronary artery [33], 29 patients (29.9%) presented CAD-RADS 0, 27 (27.8%) had 1, 24 (24.7%) had 2, 9 (9.3%) had category 3, 5 (5.1%) were diagnosed with 4 and 1 (1.0%) had 5. The calculation for 2 patients (2.1%) was not diagnostic. The obtained mean left ventricle ejection fraction was 69.58% (±8.18). The measured mean EAT parameters were 9.51 mm (±3.33) for thickness, 60.03 mL (±21.07) for volume, 5.23 mm/m^2^ (±1.83) for thickness index and 33.08 mL/m^2^ (±11.98) for volume index. The measured mean aortic diameters were 33.42 mm (±4.36) and 34.50 mm (±4.42) for diastolic and systolic, respectively. The mean aortic stiffness index was 4.18 (±0.81). The assessed mean aortic elasticity parameters were 3.29% (±2.37) and 0.12 cm^2^/dyn (±0.09) for strain and distensibility, respectively.

### 3.2. Relation between Volume and Thickness of Epicardial Adipose and Aortic Stiffness and Elasticity—Higher Thickness and Volume of EAT in Group of Higher Ao Stiffness and Lower Ao Elasticity

The detailed results are summarized in Table 3. We compared EAT mean parameters between subgroups distinguished according to aortic parameters. In the subgroup composed of participants with an aortic stiffness index equal to or greater than the median (4.09) means of EAT thickness (10.26 mm vs. 8.74 mm), volume (67.84 mL vs. 52.06 mL), thickness index (5.64 mm/m^2^ vs. 4.81 mm/m^2^) and volume index (37.66 mL/m^2^ vs. 28.40 mL/m^2^) were statistically significantly (*p* < 0.05) higher than in the subgroup with an aortic stiffness index below the median. In the subgroups analyzing EAT and aortic elasticity, we analyzed EAT in comparison to aortic strain and distensibility. In the subgroup composed of participants with aortic strain below the median (<2.67%) means of EAT thickness (10.12 mm vs. 8.89)mm, volume (67.81 mL vs. 52.10 mL), thickness index (5.57 mm/m^2^ vs. 4.88 mm/m^2^) and volume index (37.64 mL/m^2^ vs. 28.41 mL/m^2^) were higher compared to the subgroups with aortic strain equal to or greater than the median. However, only EAT volume and volume index differences were statistically significant (*p* < 0.05). Similarly, in the subgroup with aortic distensibility below the median (<0.10 cm^2^/dyn) means of EAT thickness (10.14 mm vs. 8.89 mm), volume (67.55 mL vs. 52.67 mL), thickness index (5.58 mm/m^2^ vs. 4.89 mm/m^2^) and volume index (37.48 mL/m^2^ vs. 28.76 mL/m^2^) were higher compared to the subgroups with aortic distensibility equal to or greater than the median and, similarly, once again only the EAT volume and volume index differences were statistically significant.

### 3.3. Relation between Stiffness and Elasticity of Aorta and EAT—Higher Ao Stiffness and Lower Ao Elasticity in Subgroups of Higher EAT Thickness and Volume

The detailed results are summarized in Table 4. We compared the means of Ao stiffness and elasticity parameters between the subgroups, differentiated based on the medians of EAT parameters. In the subgroups composed of participants with EAT thickness equal to or higher than median (9.40 mm), the mean of Ao stiffness index was higher (4.35 vs. 4.01) and, in contrast, the means of Ao strain (2.64% vs. 3.96%) and Ao distensibility (0.10 cm^2^/dyn vs. 0.14 cm^2^/dyn) were lower compared to the subgroups with EAT thickness below the median. However, only the Ao strain and Ao distensibility differences were statistically significant. Similarly, in the subgroup composed of participants with EAT volume equal to or greater than the median (61.95 mL), the mean of aortic stiffness was higher (4.39 vs. 3.96) and, on the contrary, the means of Ao strain (2.61% vs. 3.98%) and Ao distensibility (0.10 cm^2^/dyn vs. 0.15 cm^2^/dyn) were lower compared to the subgroup with EAT volume below the median, and all differences were statistically significant. Likewise, in the subgroup of participants with EAT thickness index equal to or greater than the median (5.08 mm/m^2^), the mean of Ao stiffness was higher (4.41 vs. 3.95) and the means of Ao strain (2.50% vs. 4.09%) and Ao distensibility (0.09 cm^2^/dyn vs. 0.15 cm^2^/dyn) were lower compared to the subgroups with EAT thickness index below the median and all differences were statistically significant. Comparably, in the subgroup including participants with EAT volume index equal to or greater than the median (34.33 mL/m^2^), Ao stiffness (4.56 vs. 3.79) and Ao strain (2.27% vs. 1.78%) were higher and Ao distensibility (0.09 cm^2^/dyn vs. 0.16 cm^2^/dyn) was lower. However, only the Ao stiffness and Ao distensibility differences were statistically significant compared to the subgroup with an EAT volume index below the median.

### 3.4. Analysis of Correlations between EAT and Aortic Parameters

The detailed results are presented in Table 5. When comparing the parameters of epicardial adipose tissue with the parameters of aortic stiffness and elasticity parameters, a positive linear correlation was observed between the EAT parameters and aortic stiffness (0.21), volume (0.51), thickness index (0.24) and volume index (0.55) and, contrary for Ao elasticity, we observed a negative linear correlation between the EAT parameters: thickness (−0.32 and −0.30), volume (−0.49 and −0.48), thickness index (−0.34 and −0.31), volume index (−0.51 and −0.49) and Aortic elasticity parameters (Ao strain and Ao distensibility, respectively).

### 3.5. Predictor of Greater Stiffness and Lesser Elasticity of Aorta

**Figure 1:**
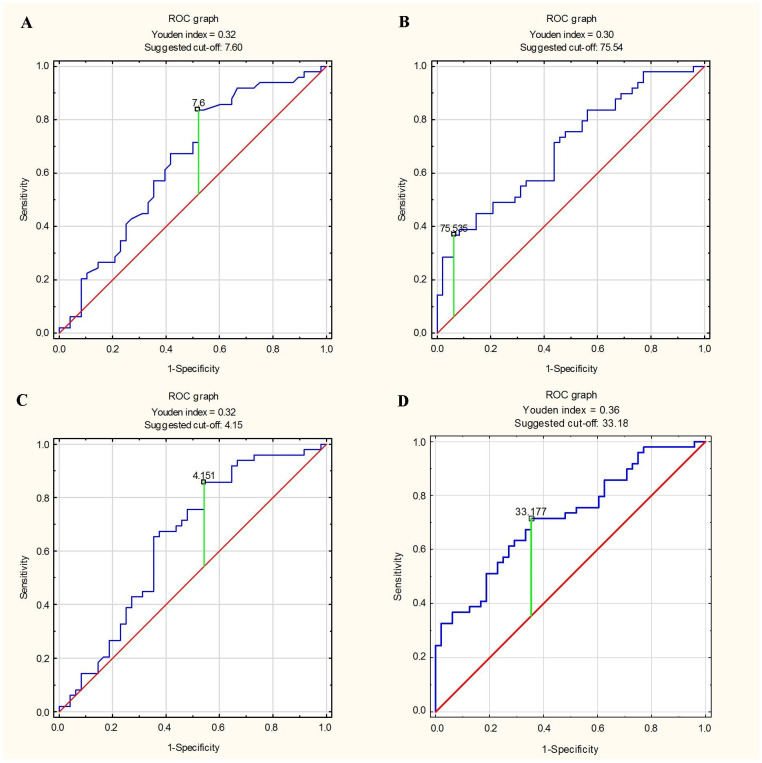

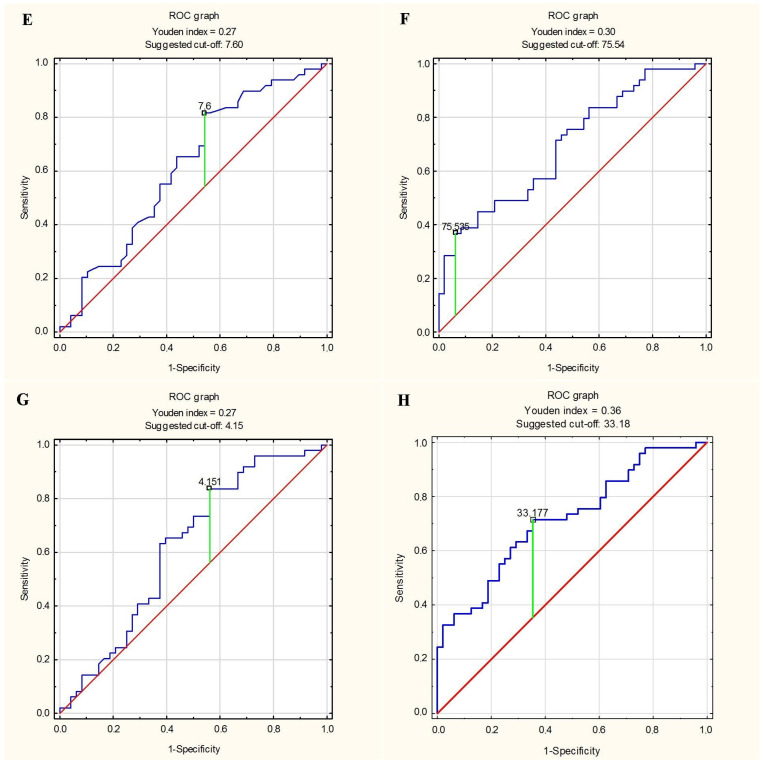

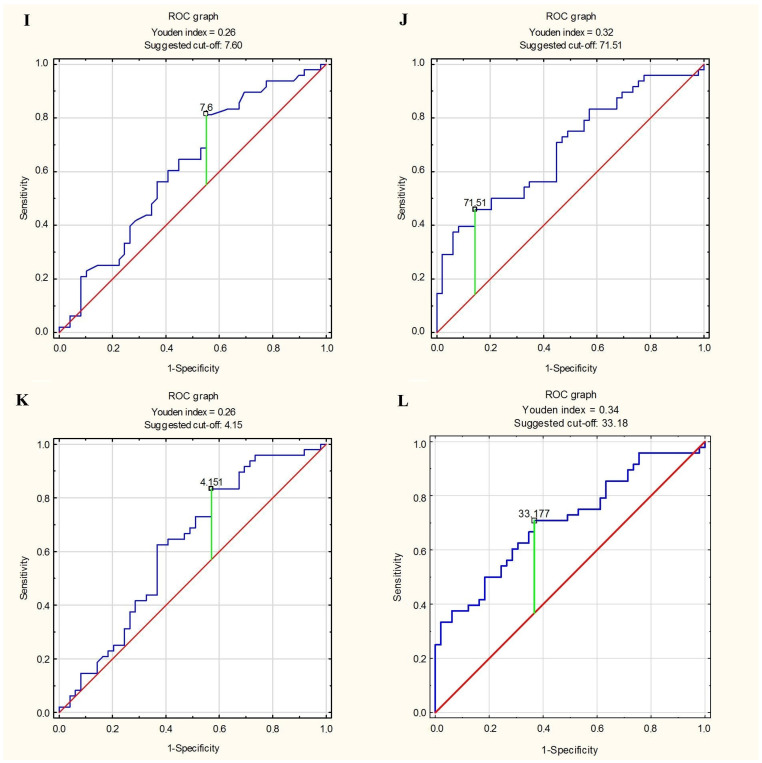
ROC curves for predicting aortic stiffness and elasticity using epicardial adipose tissue parameters. (**A**) Prediction of Ao stiffness index ≥ median using EAT thickness [mm]. (**B**) Prediction of Ao stiffness index ≥ median using EAT volume [mL]. (**C**) Prediction of Ao stiffness index ≥ median using EAT thickness index [mm/m^2^]. (**D**) Prediction of Ao stiffness index ≥ median using EAT volume index [mL/m^2^]. (**E**) Prediction of Ao strain [%] < median using EAT thickness [mm]. (**F**) Prediction of Ao strain [%] < median using EAT volume [mL]. (**G**) Prediction of Ao strain [%] < median using EAT thickness index [mm/m^2^]. (**H**) Prediction of Ao strain [%] < median using EAT volume index [mL/m^2^]. (**I**) Prediction of Ao distensibility [cm^2^/dyn] < median using EAT thickness [mm]. (**J**) Prediction of Ao distensibility [cm^2^/dyn] < median using EAT volume [mL]. (**K**) Prediction of Ao distensibility [cm^2^/dyn] < median using EAT thickness index [mm/m^2^]. (**L**) Prediction of Ao distensibility [cm^2^/dyn] < median using EAT volume index [mL/m^2^].

In Figure 1 and Table 6, we present the results of our analysis that estimated the best predictive factor of greater stiffness and lesser elasticity of aorta. During our analysis, we compared different mean parameters of EAT to Ao strain and distensibility medians, and we calculated sensitivity, specificity and accuracy. We found that, among the assessed epicardial adipose tissue variables, the EAT volume index is the best predictive factor of the mechanical properties of the aorta. The sensitivity and specificity of predicting the mechanical properties of the aorta using the EAT volume index were above 50%; such predictions were characterized by the highest accuracy.

## 4. Discussion

In our study, we found that the EAT thickness and thickness index, volume and volume index were significantly higher in the subgroup with a higher aortic stiffness index. We found that EAT volume and volume index were significantly higher in the subgroups with lower aortic strain and lower aortic distensibility. Furthermore, we found significantly lower aortic strain and distensibility in the subgroup with higher EAT thickness. We found significantly higher aortic stiffness, and lower aortic strain and aortic distensibility in the groups with higher EAT volume and EAT thickness index, and we found a significantly higher aortic stiffness index and lower aortic distensibility in the groups with higher EAT volume index. We obtained a positive correlation between EAT thickness and volume parameters and aortic stiffness index, and a negative one for Ao strain and distensibility. We also estimated that EAT volume index would be the best predictor of increased stiffness and decreased elasticity of aorta.

Terms describing epicardial adipose tissue and its parameters, and aortic stiffness and elasticity and its parameters, were used to search manuscripts in the PubMed database. Most of the relevant publications found were focused on relations between EAT and chronic diseases, and assessed the link between EAT and general arterial stiffness measured in pulse wave velocity or cardio-ankle vascular index; few studies have paid attention to comparing EAT with aortic parameters.

A study conducted by Altun et al. involved a group of approximately 60 patients who had suffered an acute ischemic stroke, and about 80 patients in control group. In this study, aortic stiffness was measured by transthoracic ultrasonography and authors calculated aortic stiffness index, strain and distensibility with the equations corresponding to those used in our study. However, the way of measuring EAT was different in Altun study, whereby the researchers used echocardiography rather than CCTA. Similarly to our results, a significant negative correlation was found between EAT thickness and aortic strain (r = −0.296 vs. −0.32; in Altun and our study, respectively) [34]. Similarly, epicardial adipose tissue was assessed in CCTA in the study of 260 Korean individuals to study its correlation with arterial stiffness. Contrary to our study, the one carried out in Korea was conducted on asymptomatic individuals (patients with known cardiovascular disease were excluded from the study). However, contrary to our study, the researchers evaluated only EAT volume and, instead of measuring aorta parameters in CCTA, had used cardio-ankle vascular index (CAVI). In our study we observed the correlation of aortic stiffness with EAT and, in a study by Hye Eun Park et al., a correlation between CAVI (a parameters of arterial stiffness) and EAT was observed [29]. In addition, in a study on 176 people (156 participants with hypertension), a correlation between epicardial adipose tissue volume and aortic stiffness, measured by pulse wave velocity (both measured by cardiac magnetic resonance), was found [35]. A similar association was observed in an analogous study on 58 participants with pulse wave velocity as a measure of aortic stiffness. In this study, healthy individuals underwent a cardiac magnetic resonance exam to determine parameters such as epicardial fat volume and aortic stiffness (assessed by aortic pulse wave velocity) to determine their relationship to body mass index and age, assessed by analyses of covariance. An association was found between age and epicardial fat volume, as well as pulse wave velocity [36].

Likewise, in a study conducted on 135 patients with heart failure with preserved ejection fraction, EAT thickness was measured by echocardiography and arterial stiffness was determined by brachial-ankle pulse wave velocity. Patients were divided based on EAT thickness into two groups (<3.55 and ≥3.55 mm—these values are different to the ones we used in our study; however, in our study, we used CCTA instead of echocardiography). Participants with greater EAT thickness were more likely to have comorbidities and their arterial stiffness was increased which corresponds to our study in which we observed higher aorta stiffness [37]. Likewise, in study on 144 patients diagnosed with hypertension, measured in echocardiography an increase in EAT thickness and an increase in aortic stiffness index were found to be associated and, similarly, to our results Ao distensibility and strain were also significantly decreased [38]. Similar results were also obtained by Kim et al. in a study in which EAT thickness was associated with arterial stiffness determined by pulse wave velocity [39]. EAT thickness was also correlated with arterial stiffness measured by pulse wave velocity in patients with rheumatoid arthritis [40]. Furthermore, Argan et al. associated EAT thickness with the risk of ascending aortic dilatation [41] and similar results were obtained in a study by Canga et al. [42]. Another study included examinations using transthoracic echocardiography to measure EAT thickness and ascending aorta inner diameters of 153 participants, suggesting that thickened EAT in patients with type 2 diabetes mellitus is associated with ascending aorta elasticity, independent of blood glucose [43].

In our study, we found EAT volume and volume index to be the best predictors of increased aortic stiffness and decreased elasticity. Although there are no identical studies that compared those parameters directly, we found meta-analysis according to which EAT volume is independently associated with coronary artery stenosis, myocardial ischemia and major adverse cardiovascular events [44]. We also found another study that found the measurement of the aortic stiffness index to be an applicable method to predict subclinical atherosclerosis [32]. In addition, in one study, higher EAT volume (>120 cm^3^) assessed in CCTA was found to be highly associated with the presence of coronary artery disease, and increasing EAT volume was a predictor of more severe CAD [45].

The strength of the research conducted here is that it is a study exploring the as yet insufficiently investigated area of the relationship between increased amount of epicardial adipose tissue (both its volume and thickness) and increased stiffness and decreased elasticity of the aorta, but there are some limitations that deserve attention. The study group is relatively small, and that is why further research with a greater number of patients are required to confirm the results obtained and provide a more generalizable result. It should also be emphasized that the study carried out was designed to determine association between variables but not causality. Therefore, several statistical tests were conducted, each of which is associated with the possibility of statistical error. In order to strengthen the level of evidence, it would be necessary to conduct more research in this area in future. However, due to the use of ionizing radiation, the methodology of the study carries limitations on the exposure of subjects to harmful agents. Therefore, this study was composed of patients with the medical indications necessary to conduct CCTA (approximately 70% of them had a suspicion of CAD), which means that the participants were a specific group in comparison to the healthy population. Exposing healthy people to ionizing radiation only for the purpose of conducting a study might not be considered ethical, therefore the repetition of this study on a completely healthy and asymptomatic group would be rather unlikely to happen. However, perhaps further research will allow the development of a method giving similar results to our study but excluding ionizing radiation, with which it will become possible to carry out an analysis of the causality relationship.

The main limitations of the study include the single-center nature of the study, the small size of the study group, the over-representation of overweight/obese patients, the lack of clinical characteristics of the subjects in terms of, e.g., lipid profile and glycemia, and the large variation in the severity of CAD in the study group, with a small percentage of patients with suspected significant stenoses in the coronary arteries.

## 5. Conclusions

Coronary computed tomography angiography shows a proportional relationship between an increased amount of epicardial adipose tissue (both its volume and thickness) and increased stiffness and decreased elasticity of the aorta. Among the parameters describing epicardial adipose tissue, we found its volume index to be the most useful predictive factor of increased stiffness and decreased elasticity of aorta.

## Figures and Tables

**Table 1:** Basic clinical characteristics of the patients.

	X	SD
age [years]	63.48	8.50
height [m]	1.67	0.07
body mass [kg]	74.16	11.03
BMI [kg/m^2^]	25.97	3.81
BSA [m^2^]	1.82	0.15
	%	n
gender		
men	49.5	48
women	50.5	49
body mass		
normal	47.4	46
overweight/obesity	52.6	51
indication to CCTA		
chronic CAD suspicion	70.1	68
chest pain	39.2	38
low intermediate CAD risk	39.1	37
numerous CAD risk factors	26.8	26
inconclusive exercise test	13.4	13
non-diagnostic exercise test	8.2	8
regional wall motion abnormalities of left ventricular	2.1	2
sudden cardiac death in the family history	1.0	1
	X	SD
SBP [mmHg]	143.20	7.78
DBP [mmHg]	88.40	5.66

BMI—body mass index; CAD—coronary artery diseases; CCTA—coronary computed tomography angiography; DBP—diastolic blood pressure, n—number; SBP—systolic blood pressure, SD—standard deviation; X—mean.

**Table 2:** Coronary computed tomography angiography parameters in the study group.

	X	SD
CACS	162.24	317.69
	n	%
significant CAD risk		
practically non-existent	31	31.9
minimal	23	23.7
mild	15	15.5
moderate	16	16.5
high	12	12.4
CAD-RADS		
0	29	29.9
1	27	27.8
2	24	24.7
3	9	9.3
4	5	5.1
5	1	1.0
N	2	2.1
LVEF [%]	69.58	8.18
EAT thickness [mm]	9.51	3.33
EAT volume [mL]	60.03	21.07
EAT thickness index [mm/m^2^]	5.23	1.83
EAT volume index [mL/m^2^]	33.08	11.98
Ao diastolic diameter [mm]	33.42	4.36
Ao systolic diameter [mm]	34.50	4.42
Ao stiffness index	4.18	0.81
Ao strain [%]	3.29	2.37
Ao distensibility [cm^2^/dyn]	0.12	0.09

Ao—aorta; CACS—coronary artery calcium score; CAD—coronary artery diseases; EAT—epicardial adipose tissue; LVEF—left ventricular ejection fraction; SD—standard deviation; X—mean.

**Table 3:** Thickness and volume of epicardial adipose tissue in subgroups distinguished according to the median value of aortic stiffness and elasticity variables.

	Ao Stiffness Index ≥ Me (≥4.09, *n* = 49)		Ao Stiffness Index < Me (<4.09, *n* = 48)		*p*
	X	SD	X	SD	
EAT thickness [mm]	10.26	3.06	8.74	3.45	<0.05
EAT volume [mL]	67.84	19.68	52.06	19.55	<0.05
EAT thickness index [mm/m^2^]	5.64	1.60	4.81	1.96	<0.05
EAT volume index [mL/m^2^]	37.66	11.63	28.40	10.53	<0.05
	Ao strain ≥ Me (≥2.67%, *n* = 48)		Ao strain < Me (<2.67%, *n* = 49)		*p*
	X	SD	X	SD	
EAT thickness [mm]	8.89	3.47	10.12	3.11	ns
EAT volume [mL]	52.10	19.58	67.81	19.69	<0.05
EAT thickness index [mm/m^2^]	4.88	1.97	5.57	1.63	ns
EAT volume index [mL/m^2^]	28.41	10.54	37.64	11.63	<0.05
	Ao distensibility ≥ Me (≥0.10 cm^2^/dyn, *n* = 49)		Ao distensibility < Me (<0.10 cm^2^/dyn, *n* = 48)		*p*
	X	SD	X	SD	
EAT thickness [mm]	8.89	3.43	10.14	3.13	ns
EAT volume [mL]	52.67	18.90	67.55	20.68	<0.05
EAT thickness index [mm/m^2^]	4.89	1.95	5.58	1.64	ns
EAT volume index [mL/m^2^]	28.76	10.20	37.48	12.15	<0.05

Ao—aorta; EAT—epicardial adipose tissue; Me—median; ns—non-statistically significant; SD—standard deviation; X—mean.

**Table 4:** Aortic stiffness and elasticity parameters in subgroups distinguished according to the median value of epicardial adipose tissue variables.

	EAT Thickness ≥ Me (≥9.40 mm, *n* = 49)		EAT Thickness < Me (<9.40 mm, *n* = 48)		*p*
	X	SD	X	SD	
Ao stiffness index	4.35	0.71	4.01	0.87	ns
Ao strain [%]	2.64	1.84	3.96	2.66	<0.05
Ao distensibility [cm^2^/dyn]	0.10	0.07	0.14	0.10	<0.05
	EAT volume ≥ Me (≥61.95 mL, *n* = 49)		EAT volume < Me (<61.95 mL, *n* = 48)		*p*
	X	SD	X	SD	
Ao stiffness index	4.39	0.78	3.96	0.79	<0.05
Ao strain [%]	2.61	1.82	3.98	2.67	<0.05
Ao distensibility [cm^2^/dyn]	0.10	0.07	0.15	0.11	<0.05
	EAT thickness index ≥ Me (≥5.08 mm/m^2^, *n* = 49)		EAT thickness index < Me (<5.08 mm/m^2^, *n* = 48)		*p*
	X	SD	X	SD	
Ao stiffness index	4.41	0.72	3.95	0.84	<0.05
Ao strain [%]	2.50	1.85	4.09	2.58	<0.05
Ao distensibility [cm^2^/dyn]	0.09	0.07	0.15	0.10	<0.05
	EAT volume index ≥ Me (≥34.33 mL/m^2^, *n* = 49)		EAT volume index < Me (<34.33 mL/m^2^, *n* = 48)		*p*
	X	SD	X	SD	
Ao stiffness index	4.56	0.81	3.79	0.60	<0.05
Ao strain [%]	2.27	4.33	1.78	2.46	ns
Ao distensibility [cm^2^/dyn]	0.09	0.07	0.16	0.09	<0.05

Ao—aorta; EAT—epicardial adipose tissue; Me—median; ns—non-statistically significant; SD—standard deviation; X—mean.

**Table 5:** Correlations between parameters of epicardial adipose tissue and parameters of aortic stiffness and elasticity.

	Ao Stiffness Index	Ao Strain [%]	Ao Distensibility [cm^2^/dyn]
EAT thickness [mm]	0.21	−0.32	−0.30
EAT volume [mL]	0.51	−0.49	−0.48
EAT thickness index [mm/m^2^]	0.24	−0.34	−0.31
EAT volume index [mL/m^2^]	0.55	−0.51	−0.49

Ao—aorta; EAT—epicardial adipose tissue.

**Table 6:** Sensitivity, specificity and accuracy of parameters of epicardial adipose tissue as aortic stiffness and elasticity predictors.

Prediction Conditions		Sensitivity	Specificity	Accuracy
Predictive Factor	Real State			
EAT thickness ≥ 7.6 mm	Ao stiffness index ≥Me (≥4.09)	0.479	0.837	0.660
EAT volume ≥ 75.54 mL	Ao stiffness index ≥Me (≥4.09)	0.938	0.347	0.639
EAT thickness index ≥ 4.15 mm/m^2^	Ao stiffness index ≥Me (≥4.09)	0.458	0.857	0.660
EAT volume index ≥ 33.18 mL/m^2^	Ao stiffness index ≥Me (≥4.09)	0.646	0.694	0.670
EAT thickness ≥ 7.6 mm	Ao strain <Me (<2.67%)	0.458	0.816	0.639
EAT volume ≥ 75.54 mL	Ao strain <Me (<2.67%)	0.938	0.347	0.639
EAT thickness index ≥ 4.15 mm/m^2^	Ao strain <Me (<2.67%)	0.438	0.837	0.639
EAT volume index ≥ 33.18 mL/m^2^	Ao strain <Me (<2.67%)	0.646	0.694	0.670
EAT thickness ≥ 7.6 mm	Ao distensibility <Me (<0.10 cm^2^/dyn)	0.449	0.813	0.629
EAT volume ≥ 71.51 mL	Ao distensibility <Me (<0.10 cm^2^/dyn)	0.857	0.438	0.649
EAT thickness index ≥ 4.15 mm/m^2^	Ao distensibility <Me (<0.10 cm^2^/dyn)	0.429	0.833	0.629
EAT volume index ≥ 33.18 mL/m^2^	Ao distensibility <Me (<0.10 cm^2^/dyn)	0.633	0.633	0.660

Ao—aorta; EAT—epicardial adipose tissue.

## Data Availability

Study data can be made available upon documented request.
